# Incidental detection of ectopic liver tissue during elective cholecystectomy: a case report

**DOI:** 10.3389/fsurg.2025.1565209

**Published:** 2025-05-16

**Authors:** Ahmet Cihangir Emral

**Affiliations:** Department of General Surgery, Atılım University Faculty of Medicine, Ankara, Türkiye

**Keywords:** ectopic liver tissue, gallbladder, laparoscopic cholecystectomy, malignant transformation, hepatocellular carcinoma

## Abstract

**Introduction:**

Ectopic liver tissue (ELT) is a rare embryological anomaly most commonly found in the abdominal region, particularly on the gallbladder. It is generally asymptomatic and is often discovered incidentally during surgery or autopsy. Although histologically similar to the main liver, ELT should be excised due to the potential risk of malignancy.

**Case presentation:**

This study presents a case of incidental ELT discovered during elective cholecystectomy in a 34-year-old female patient. Preoperative imaging revealed multiple gallstones, and during laparoscopic exploration, an ectopic liver tissue lesion was found on the gallbladder fundus. The lesion, measuring 10 × 5 mm, was excised *en bloc* with the gallbladder and sent for pathological examination, which showed no malignancy.

**Discussion:**

The rarity and asymptomatic nature of ELT make it difficult to diagnose preoperatively, but its association with malignant transformation warrants careful management. In cases of suspected malignancy, further surgical approaches such as extended surgical margins and regional lymph node dissection should be considered.

**Conclusion:**

This case underscores the importance of early detection and excision of ectopic liver tissue due to its potential for malignant transformation.

## Introduction

Ectopic liver tissue (ELT) is most commonly found in the abdominal region, particularly on the gallbladder, as well as in the gastrohepatic or umbilical ligament, omentum, stomach, pancreas, and spleen (1). ELT is generally small in size and asymptomatic. The formation of ELT tissue is hypothesized to be due to disruptions in the normal migratory patterns of hepatic cells during development, while pedunculated forms may emerge from the abnormal overgrowth of hepatic cords. Unlike accessory liver tissue, ectopic liver tissue is not connected to the main liver. Although histologically similar to the main liver, ectopic liver tissue should be excised due to the increased risk of malignancy (1, 2). It was first reported by Corsy et al. in 1922 (3). Despite advancements in preoperative radiological imaging techniques and their increasing usage, reported cases of ectopic liver tissue are typically discovered incidentally during surgery and/or autopsy (2, 3). In this study, we present a case of incidental ectopic liver tissue detected during elective cholecystectomy.

## Case presentation

**Figure 1:**
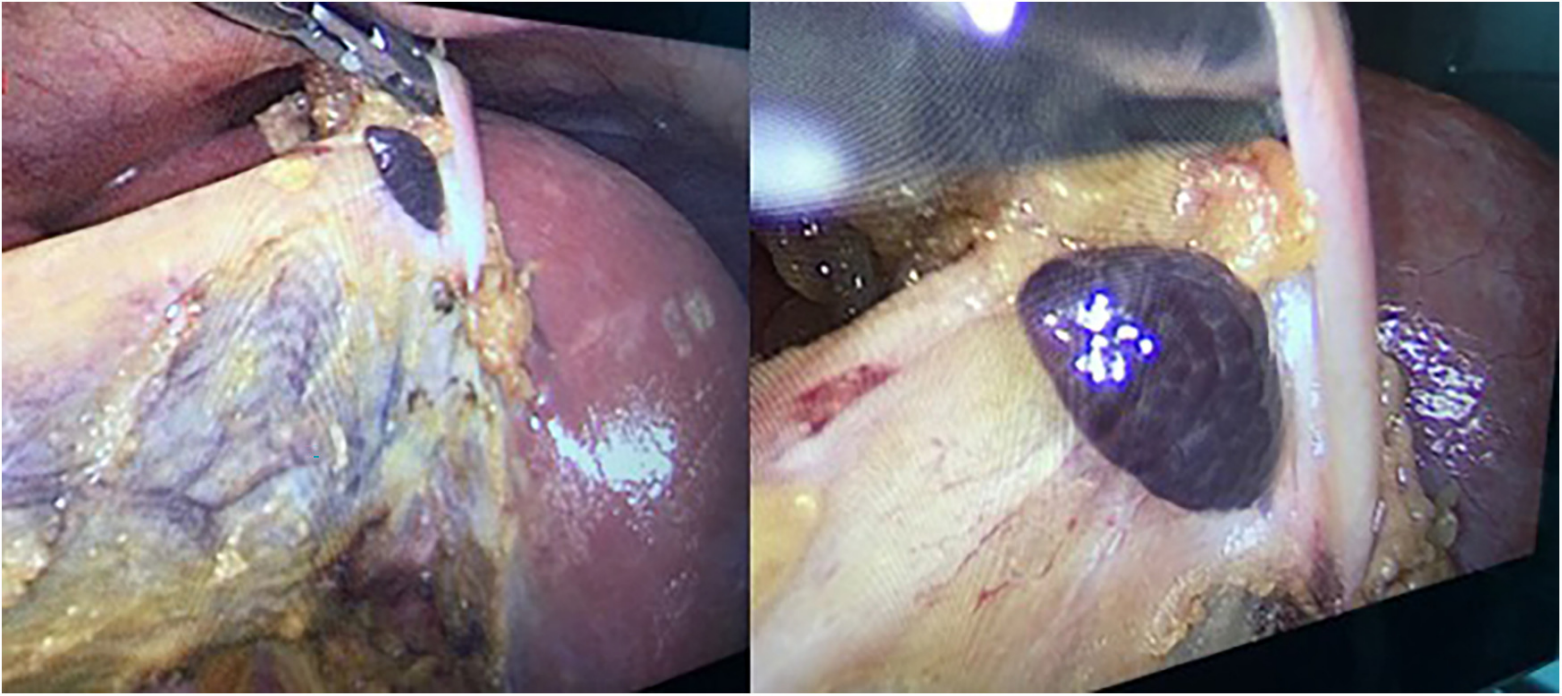
Ectopic liver tissue observed incidentally on the gallbladder during laparoscopic cholecystectomy.

**Figure 2:**
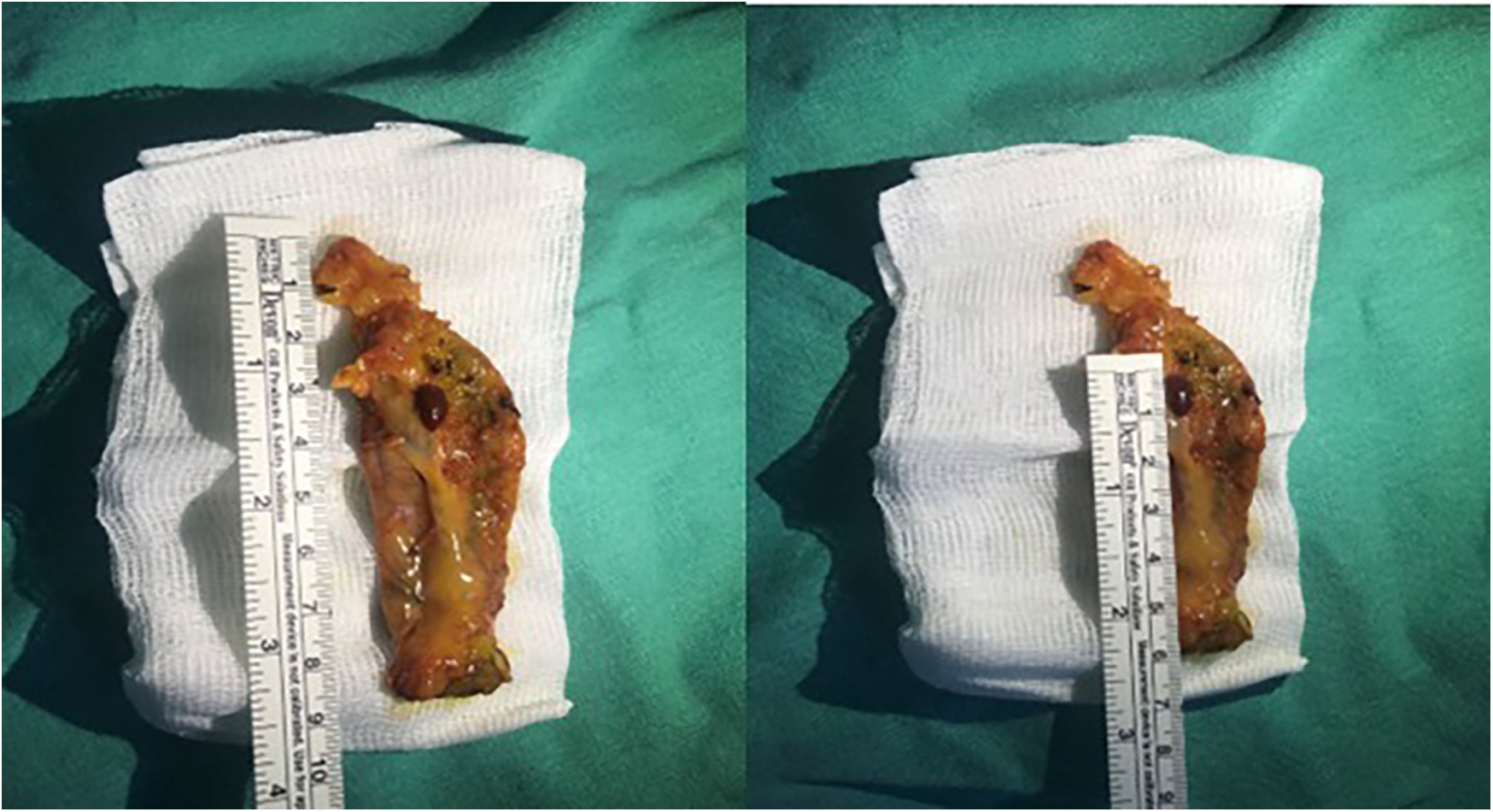
Macroscopic view of the specimen and its dimensions.

**Figure 3:**
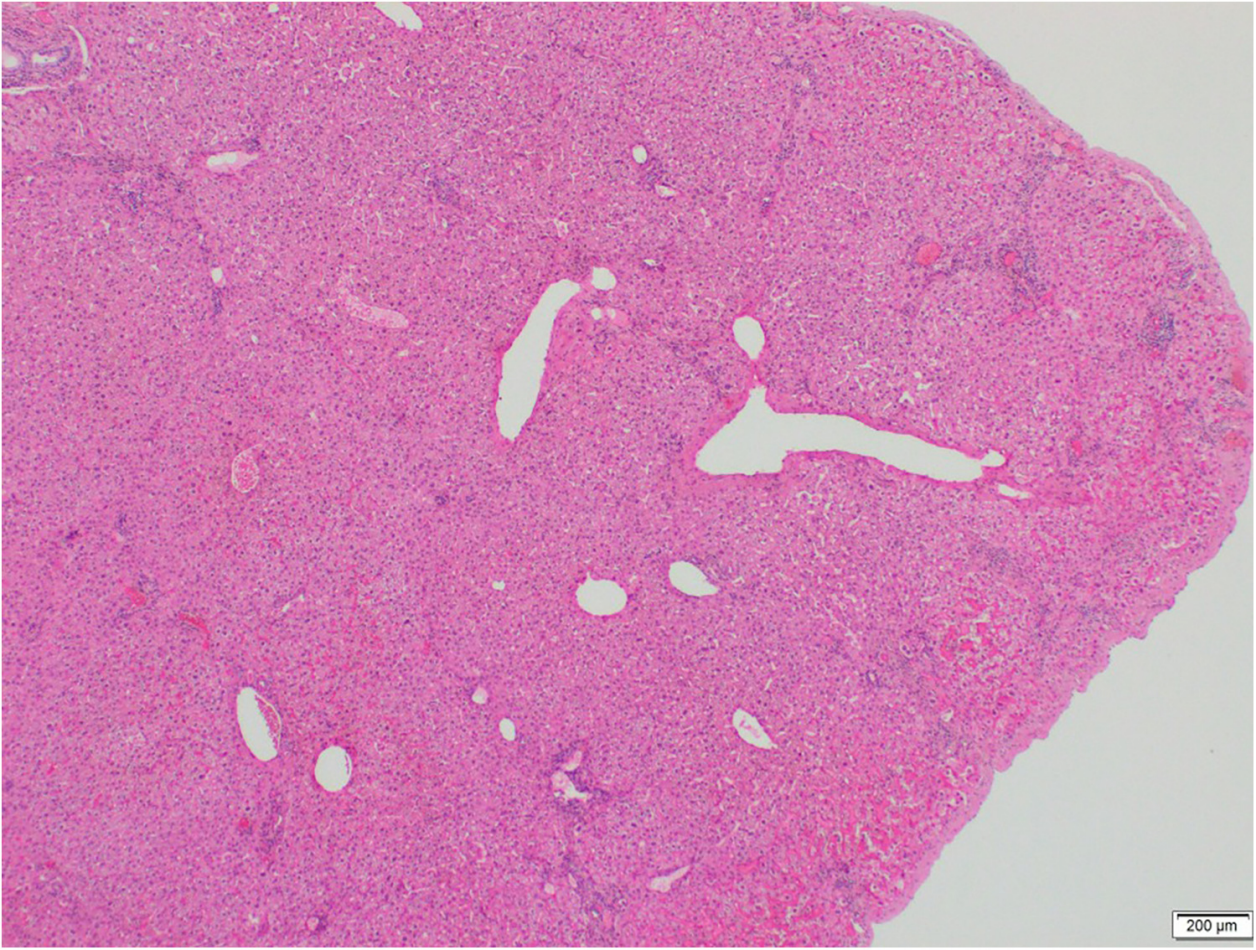
The ectopic liver demonstrates normal microanatomical histology. The portal region and hepatocytes are clearly visible. No nodular configuration, significant fibrosis, bridging necrosis, or lobular inflammation is observed. (H&E stain, ×40).

A 34-year-old female patient presented with a one-year history of dyspeptic symptoms and intermittent pain in the right upper quadrant, particularly after consuming fatty foods. The patient had no prior surgical history and no relevant systemic disease history. Preoperative physical examination and laboratory results were normal. Preoperative ultrasound revealed multiple gallstones, the largest of which measured approximately 1 cm in size. Elective cholecystectomy was planned for the patient. During laparoscopic exploration, a tissue lesion, consistent with liver and spleen tissue, was observed on the gallbladder fundus. This lesion was not connected to the main liver (Figures 1, 2). A laparoscopic cholecystectomy was performed, and the lesion, along with the gallbladder, was excised *en bloc* through the umbilical port site in an endobag. The ectopic liver tissue measured 10 × 5 mm and was sent for pathological examination under appropriate conditions. The pathological examination revealed no signs of malignancy (Figure 3).

## Discussion

Due to the rarity of ectopic liver tissue (ELT), its incidence remains unclear, with a reported prevalence ranging between 0.24% and 0.47% in the literature (3). Unlike accessory liver tissue, ectopic liver tissue is characterized by the absence of a connection to the main liver. ELT may be associated with congenital anomalies such as biliary atresia, caudate lobe agenesis, omphalocele, bile duct cysts, and cardiac abnormalities (1, 4). Although most commonly found on the gallbladder, ectopic liver tissue has also been reported in other locations, including the adrenal glands, pancreas, spleen, falciform ligament, stomach-pylorus, umbilicus, retroperitoneum, thorax (intra- and extra-pleural), and pericardium (5). In a literature review by Akbulut et al., 91 patients (62 female, 25 male, 4 with unknown sex) with ectopic liver tissue were examined. According to this study, the mean size was found to be 17.8 mm (4).

ELT is generally asymptomatic. Symptomatic patients are usually infants, with abdominal pain being the most common complaint. Abdominal pain may arise from complications such as torsion, intraperitoneal hemorrhage, pyloric obstruction, and rupture (1, 5). In addition, Leone et al. (6) demonstrated in their studies that in patients with ectopic liver tissue (ELT) developing HCC, particularly those with large tumors, laboratory findings of anemia, clinical signs of intraperitoneal hemorrhage, and changes due to compression effects on other organs were observed. Ectopic liver like the main liver tissue can undergo fatty changes, hemosiderosis, cholestasis, cirrhosis, hepatitis, or malignant degeneration to hepatocellular carcinoma (4). Furthermore, ELT can present as an unwanted disease when it leads to pressure symptoms following the development of hepatocellular carcinoma (HCC). Although histologically similar to the main liver tissue, the structure of ectopic liver tissue is not fully functional, leading to metabolic impairment. This is why malignant transformation is thought to be more likely in these tissues (7, 8). In a review by Yamashita et al. (9), 70 patients with ectopic liver tissue were reported, with one (4.5%) of 22 patients having ectopic liver tissue attached to the gallbladder developing hepatocellular carcinoma (HCC), while eight (16.7%) of 48 patients with ectopic hepatic tissue located outside the gallbladder developed HCC.

In the study by Arakawa et al. (8), two patients with ectopic liver were described, one of whom developed hepatocellular carcinoma (HCC) in the ectopic tissue. In addition, their review of the literature identified more than 20 reported cases of HCC arising in ectopic liver tissue, even in the absence of cirrhosis in the main liver. This suggests that ectopic liver tissue can develop HCC much earlier than the main liver. Interestingly, when comparing the development of HCC in ectopic liver tissue found in the gallbladder with that in other locations, it was found that the incidence of HCC was lower in cases involving the gallbladder (8).

The rarity, small size, and asymptomatic nature of ectopic liver tissue contribute to a lack of awareness among radiologists. Consequently, it is often not diagnosed during preoperative radiological investigations (especially ultrasound). If diagnosed or incidentally discovered, it is recommended to excise the tissue *en bloc* in an endobag, as it may contain malignancy (2, 3, 10). If histopathological examination reveals the presence of HCC in the ectopic liver tissue, a second surgical approach with extended surgical margins and regional lymph node dissection should be considered (10, 11).

## Conclusion

Ectopic liver tissue is considered a rare embryological anomaly. Due to its usually asymptomatic nature, incidental detection, and potential presence in various locations, it requires careful consideration. Given the increased risk of malignant transformation, ectopic liver tissue should always be excised and subjected to histopathological examination.

## Data Availability

The original contributions presented in the study are included in the article/Supplementary Material, further inquiries can be directed to the corresponding author.
